# *In Vitro* Seed Germination and Seedling Development of a Rare Indonesian Native Orchid *Phalaenopsis amboinensis* J.J.Sm

**DOI:** 10.1155/2019/8105138

**Published:** 2019-02-03

**Authors:** Edy Setiti Wida Utami, Sucipto Hariyanto

**Affiliations:** Department of Biology, Faculty of Science and Technology, Universitas Airlangga, Surabaya 60115, Indonesia

## Abstract

*Phalaenopsis amboinensis*, an epiphytic orchid, has a great potential for commercial exploitation in the cut-flower industry. It is difficult to propagate vegetatively as it naturally grows slowly. Therefore, there is a need to improve the propagation methods to avoid endangering its natural populations. The objective of this study was to identify the best medium and organic supplements for seed germination and plantlets development of *P. amboinensis*. Seeds from 4-month-old hand-pollinated orchids were sown on different strengths of MS and VW culture media: Murashige and Skoog (MS), 1/2 MS, Vacin and Went (VW), and 1/2 VW. Optimum seed germination, i.e., 90.7%, was achieved on VW medium. VW medium was suitable for seedling formation and allowed 51.4% of seedling development from protocorm within 10 weeks of culture. When 15% (v/v) coconut water was added together with banana homogenate (10 g·L^−1^) to the VW medium, the plantlets grew to the highest length and had the highest dry weight (62.1 mm and 15.5 g, respectively). The roots and leaves of the plantlets grew vigorously in this medium. Plants regenerated via *in vitro* seed germination processes were successfully acclimatized in greenhouse conditions, and the survival rate was more than 85%.

## 1. Introduction

*Phalaenopsis*, the moth orchid, has been recognized as one of the most beautiful flowers in the world [1]. This genus has economic value for pot plant and cut-flower production. However, it is difficult to propagate vegetatively as it naturally grows slowly [2]. *P. amboinensis* is an epiphytic monopodial orchid of tropical origin and is found at an elevation of 500–700 m. *P. amboinensis* from Sulawesi has yellow flowers with brown fine lines, whereas the white-based flowers with brown or reddish brown thick lines are found in Maluku.

*Phalaenopsis amboinensis* is a native species from Indonesia which has a great potential for commercial exploitation in cut-flower industry and is a parent for orchid breeding. The product of plant breeding *of P. amboinensis* with *P. amabilis* could potentially produce a greater flower and prettier color than both parents. The plant breeding *of P. amboinensis* with *P. violacea* produced the hybrid *P.* Princess Kaiulani, whereas *P. amboinensis* and *P. fasciata* generated the hybrid *P.* Golden Pride. Furthermore, *P. amboinensis* with *P. venosa* produced the hybrid *P.* Ambonosa, whereas breeding of *P. amboinensis* and *P. mannii* generated the hybrid *P.* Mambo. However, the existence of this orchid in native habitat is endangered due to the overexploitation and deforestation. *P. amboinensis* is one of the endemic orchid species protected by the government of Indonesia to conserve the endemic orchid from extinction. The techniques could be conducted by *in situ* or *ex situ* conservation. *Ex situ* conservation could be executed in a botanical garden, orchid nursery, and university. *In vitro* seed culture is a proper method to propagate the endemic or threatened plant species for conservation in order to maintain variability of plant genetics.

Lots of propagation techniques were developed for *Phalaenopsis* species and their hybrids through the *in vitro* culture from various explants, including leaf segments/tissue [3], flower stalk nodes [4], callus and protocorm-like bodies [5], flower stalks [6], and seeds [7, 8]. However, propagation through *in vitro* seed culture has not yet been achieved in *P. amboinensis* orchid.

The success of seed germination through *in vitro* culture is influenced by several factors, including the types of culture media [9, 10], seed maturity [11, 12], plant growth regulators [13, 14], carbohydrates [15], and organic amendments [16].

In the present study, we evaluated the effects of different strengths of MS and VW media on seed germination and supplementation of coconut water with various concentrations of banana homogenate on the plantlet development of *P. amboinensis*. The reproducible protocol for the production of seedlings from seeds by *in vitro* culture of this species has been established.

## 2. Materials and Methods

### 2.1. Plant Material, Surface Sterilization of Capsules, and Collection of Seeds

**Figure 1:**
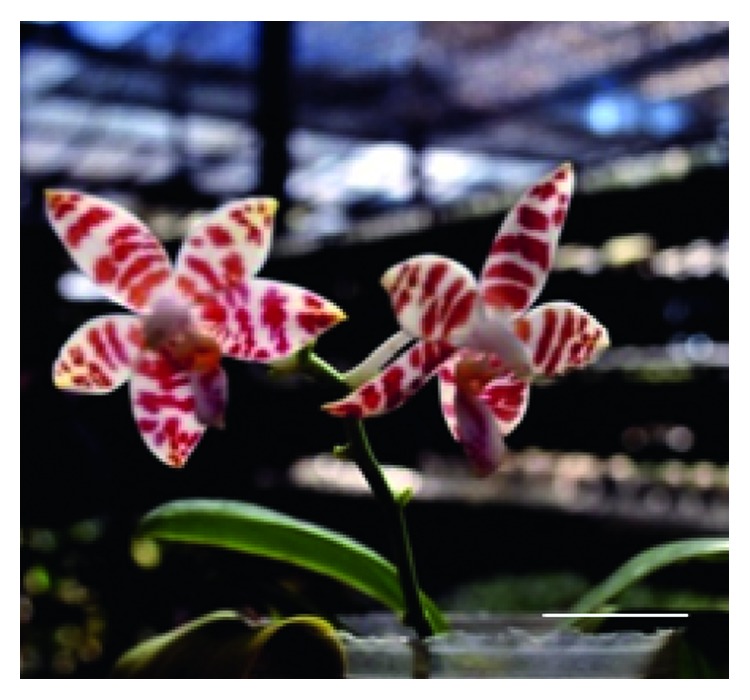
Flowering plant of *Phalaenopsis amboinensis* J.J.Sm. Scale bars: 3 cm.

*Phalaenopsis amboinensis* J.J.Sm. (Maluku) (Figure 1) was kindly provided by Handoyohardjo Orchids Nursery, East Java, Indonesia. The yellowish green capsules, which are four-month old from hand pollinating, were washed with soapy solution (10 v/v) of Sunlight (commercial detergent, Unilever, Indonesia) for 5 minutes to eliminate the dust, and then it was rinsed 3 times with sterile-distilled water and put on a Petri dish in a laminar flow hood followed by flaming 3 times. The surface-sterilized capsules were slit longitudinally into two sections with a sterile surgical blade in a sterile Petri dish to isolate the seeds.

### 2.2. *In Vitro* Germination on Different Basal Media

To evaluate the influence of different strengths of MS and VW media on seed germination and protocorm development, the seeds were sown on four media: (1) Vacin and Went (VW) [17], (2) Murashige and Skoog (MS) [18], (3) half-strength MS (1/2 MS macro- and micronutrients), and (4) half-strength VW (1/2 VW macro- and micronutrients). All media were supplemented with 30 g·L^−1^ sucrose (Merck, made in Germany) and 3 g·L^−1^ peptone (Difco Laboratories Detroit, USA) and solidified with 2 g·L^−1^ gellan gum (Phytagel; Sigma Chemical Co., St. Louis, MO). The pH was adjusted to 5.6 with 0.1 M KOH or HCl before the addition of gellan gum. Media were sterilized at 120°C for 15 minutes. Each treatment contained approximately 300 seeds, which were cultured in culture tube loaded with 25 mL of medium. All experiments consisted of three independent replicates with four culture tubes per replicate. All the cultures were maintained under a 16 and 8 h light and darkness, respectively, at 23 ± 2°C.

### 2.3. Seed Morphological Observation

The samples (seeds) were fixed in 2% (v/v) glutaraldehyde in 0.1 M phosphate buffer (pH 7.0) at 4°C for 12 h. The samples were washed in the phosphate buffer at 4°C for 3 times and dehydrated in the ethanol series followed by drying at critical point, affixing to aluminium stubs, and finally coating with gold palladium. The prepared samples were examined and photographed with a JSM-T100 scanning electron microscope (Jeol, Tokyo, Japan).

### 2.4. Histological Analysis

The seeds were fixed in FAA (70% ethyl alcohol : glacial acetic acid : formaldehyde, 90 : 5 : 5 v/v/v) and dehydrated in ethyl alcohol series, and it was then embedded in paraffin wax for 24 h. Followed by the use of a rotary microtome (Shibuya, Japan) to make longitudinal sections at 10 *µ*m thickness, stain with 1.0% safranin and 1.0% fast green and then mounted with Canada Balsam Synthetic in Xylene (Aldon, USA). Seed histology was observed in light microscope (Olympus FSX100, Japan). Seed and embryo size (length and width) were measured (at the longest and widest axis) using a light microscope with a micrometer. The data length and width were collected in 30 replicates.

### 2.5. *In Vitro* Plantlet Development

After 10 weeks of culture, seedlings (0.5 cm long with 1-2 leaves and one root) obtained from the *in vitro* seed germination were cultured individually on the fresh VW medium, supplemented with CW 15% (v/v) at various BH concentrations (0, 5, 10, and 15 g·L^−1^), and medium without BH and CW supplementation was used as the control treatment. Each treatment (4 seedlings) was planted in culture tube loaded with 25 mL of medium. All experiments consisted of three independent replicates with five culture tubes per replicate. All the cultures were maintained under a 16 and 8 h light and darkness, respectively, at 23 ± 2°C. After 10 weeks, the number and length of leaves, roots, and plantlet, the max width of leaves, and dry weight of plantlet were recorded.

### 2.6. *Ex Vitro* Plant Acclimatization

The healthy *in vitro* plantlets with well-developed roots were removed from the culture tube, washed under running tap water, and transplanted to the plastic pots containing a mixture of coal pieces : tree fern roots : sphagnum moss at 1 : 1 : 1 ratio. Potted plants were grown in the green house under 30%–40% natural light and sprayed twice a day with water for acclimatization. In this experiment, 250 plantlets were transplanted in plastic pots. Every plastic pot was loaded with 5 plantlets. The percentage of plantlets survival was recorded 12 weeks after transplanting, and plantlets survival percent was calculated using the following formula:

(1) $$\begin{array}{c} \frac{\text{no}.\text{ of plantlets survival}}{\text{no}.\text{ of plantlets transplanted}}\times 100. \end{array}$$

### 2.7. Experimental Design, Data Collection, and Analysis

**Figure 2:**
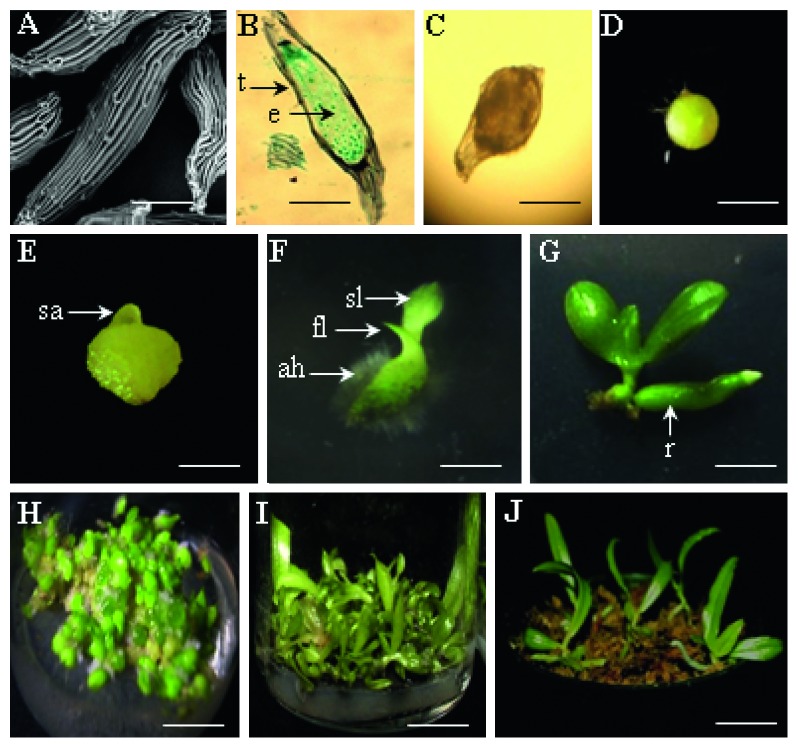
*In vitro* seed culture and propagation of *P. amboinensis* J.J.Sm. (A) Mature seed under scanning electron microscopy; (B) stage 0, no germination, long section of viable seed in elliptical embryo shape; (C) stage 1, germination, swollen embryo; (D) stage 2, embryo is completely discharged from the testa; (E) stage 3, embryo with pointed shoot apex; (F) stage 4, appearance of absorbing hair and emergence of leaves; (G) stage 5, seedling stage, root with leaves; (H) *in vitro* seed culture and protocorm development on VW medium eight weeks after inoculation; (I) seedlings *in vitro* on VW medium supplemented with coconut water 15% (v/v) and banana homogenate 10 g·L^−1^; (J) plantlet development on 12 weeks after transplanted to plastic pots. e: embryo; t: testa; sa: shoot apex; ah: absorbing hair; fl: first leaf; sl: second leaf; r: root. Scale bars: (A, B) 100 *μ*m, (C) 140 *μ*m, (D) 840 *μ*m, (E) 1900 *μ*m, (F) 3.6 mm, (G) 0.9 mm, (H, I) 1.2 cm, and (J) 1.4 cm.

The experimental units were set up in a completely randomized design (CRD). Data were subjected to analysis of variance (ANOVA), and the mean values were separated using Duncan's multiple range test (DMRT) with level of significance at *α* = 0.05 [19]. The statistical package SPSS (Version 20) was used for analysis. Seed germination and protocorm development were observed at 10 weeks after inoculation by a Tension stereomicroscope (Nikon SMZ-1, Japan). The process of seed germination to seedling formation was classified into six groups of embryos development stages (Table 1), which were adapted from Steward and Zettler [20]. Seeds were considered germinated only if the swollen embryo occurred (stage 1, Figure 2(C)). Percentage of seed germination and protocorm development for each treatment were calculated by dividing the amount of seeds in each developmental stage by the total amount of seeds × 100.

## 3. Results and Discussion

### 3.1. Seed Germination and Seedling Formation

The morphological development stages of *P*. *amboinensis* from embryo to seedling were documented (Figures 2(A)–2(G)). Seed germination of *P. amboinensis* started with swelling embryo and rounding up at about 3 weeks after sowing (WAS) (Figure 2(C)). Six WAS, embryos were discharged from the testa and developed into a round, yellow form protocorm (Figure 2(D)). A shoot apex became visible at one side of the protocorm (Figure 2(E)). When the size of the protocorm became bigger about 3.6 mm, the protocorm developed into elongated shape and the color changed into green, followed by the absorbing hair and first and second leaves formation, respectively (Figure 2(F)). Finally, the roots emerged from the seedling 9 WAS (Figure 2(G)). We found that, during the developmental process, protocorm has changed from round to perfectly round and finally to elongated shape.

### 3.2. Seed Morphological Observation and Histological Analysis

The mature seeds of *P. amboinensis* which were used as explant source of this study are fusiform in shape and dark brown in color (Figures 2(A) and 2(C)). The seeds were very small, 0.349 ± 0.006 mm in length and 0.058 ± 0.001 mm in width. Cellular organization of seed was also simple. It consisted only of an undifferentiated mass of embryo cells, without endosperm, covered by testa (Figure 2(B)). According to Molvray and Chase [21] and Arditti and Ghani [22], the size of very small seeds was one of the most distinctive characteristics in the family of Orchidaceae, so it was called “dust seeds.” Like seeds, the embryos were also minutes in *P. amboinensis*. Embryo size was 0.202 ± 0.004 mm in length, 0.048 ± 0.001 mm in width, located in centre, and elliptical in shape. Arditti and Ghani [22] and Arditti and Ernst [23] reported that orchid embryo was simple and generally oval or spherical in shape; some consisted of only few cells and most of them had no endosperm.

The embryo in the seeds of orchids generally occupies a very small portion of the seed, but in this study, *P. amboinensis* had the large embryo and it occupied a major part of the seed (Figure 2(B)). The large embryo occupying a major part of the seed was also discovered in *Bulbophyllum rothschildianum* [24].

### 3.3. Effect of Different Strengths of MS and VW Media on Seed Germination and Protocorm Development of *P. amboinensis* J.J.Sm

The result of seed germination (Table 2) showed that *P. amboinensis* seed could germinate in all tested media. VW medium gave the highest percentage of germination (97.3%) compared with 1/2 VW (90.7%), 1/2 MS (89.3%), and MS (85.3%).

The result of protocorm growth (Table 2) showed that all tested media supported the protocorm growth. The VW medium was the best for protocorm growth, giving rise to 51.4% protocorm development to stage 5 of seedling significantly higher than those of 1/2 VW (35.3%), 1/2 MS (23.9%), and MS (12.9%). Therefore, the VW medium was the most effective for seed germination and protocorm growth of *P. amboinensis*. The result showed that the VW medium contained higher P level (3.77 mM) than other media 1/2 VW (1.88 mM), MS (1.26 mM), and 1/2 MS (0.63 mM), respectively. This is in conformity with the previous reports that high P concentration in VW medium promoted seed germination in *Vanda teres* [25] and seedling development in *Bulbophyllum nipondhii* [26]. The medium type and strengths influenced significantly toward germination and growth of *P. amboinensis* protocorm. Suzuki et al. [12] also reported that the germination and protocorm development were greatly influenced by different culture media among different species. It should be noted that, different orchid might prefer different types of nutritional support. Mahendran et al. [27] reported a higher percentage of germination in epiphytic orchids *Cymbidium bicolor* on LO medium than KCM, KC, and MS media. Pradhan et al. [28] reported that MS was the most suitable for germination of *Cymbidium aloifolium* rather than KC, PM, and VW media, whereas dos Santos et al. [29] working with *Brasiliorchis picta*, an epiphytic orchid, found WPM to be significantly higher than KC, 1/2 MS, and MS media. Roy et al. [30] claimed that Phytamax medium was the most suitable for germination and protocorm development of *Vanda coerulea* than Vacin and Went, MS, and Knudson C media.

### 3.4. Effect of 15% (v/v) CW Supplementation with Various Concentrations of Banana Homogenate (BH) on the Plantlet Development of *P. amboinensis* J.J.Sm

Growth and development of plant tissues, *in vitro*, could be enhanced through the addition of various organic supplements such as apple juice, tomato juice, tryptone, peptone, coconut water, banana homogenate, potato homogenate, corn extract, yeast extract, and casein hydrolysate [31–34]. In this study, the organic supplementations with CW and BH were assayed for their effectiveness in the plantlet development of *P. amboinensis.*

We have found that the organic supplementation of CW, together with BH, had profound effects upon the growth and development of *P. amboinensis* in terms of root elongation, leaf size, and plantlet elongation (Table 3). Coconut water contains sugars, amino acids, vitamins, enzymes, and organic acids [35]. It moreover contains kinetin [36] which has a cytokinin function and enhances the explant growth and regeneration by inducing cell division. Banana contains carbohydrate, mineral, amino acids, fatty acids, niacin, vitamins, cellulose, polyols, and sterols which are beneficial for further development of plantlets [37]. Arditti [38] reported that BH was a rich source of natural cytokinins, commonly added to orchid media, to stimulate the differentiation and growth of shoots at later stages. Zeng et al. [39] found that leaf size and shoot height of *Paphiopedilum wardii* increased significantly when 100 g·L^−1^ BH and 1 g·L^−1^ peptone were added on Hyponex NO26 medium. Moreover, Wu et al. [40] observed that the growth of plantlet of *Renanthera imschootiana* increased when 100 g·L^−1^ BH, 10% (v/v) CW, and 1 g·L^−1^ peptone were added to 1/4 MS medium during the culture period. Chen et al. [41] found that the addition 10 g·L^−1^ BH and 1 mg·L^−1^ NAA to a medium during the culture period was the most effective toward the seedling formation of *Paphiopedilum spicerianum.*

### 3.5. *Ex Vitro* Plant Acclimatization

After 12 weeks of transplantation, plantlets were successfully acclimatized to greenhouse conditions, and the survival rate was more than 85%.

## 4. Conclusion

The results of this study allow the establishment of a protocol for *in vitro* propagation of *P. amboinensis* J.J.Sm. Vacin and Went medium is recommended for the *in vitro* germination and protocorm development. The seedlings exhibited vigorous growth and root development on VW medium with the addition of CW 15% (v/v) + BH 10 g·L^−1^. This protocol is an efficient means for the large-scale propagation of *P. amboinensis*, which may be applicable for other *Phalaenopsis* species.

## Figures and Tables

**Table 1:** Seed germination and seedling formation stages of *P. amboinensis* (modified from [20]).

Stage	Description
0	No growth of embryo occurs and testa was found intact
1	Swollen embryo (=germination)
2	Embryo is completely discharged from the testa (=protocorm)
3	Embryo with pointed shoot apex
4	Absorbing hairs are formed on the protocorm surface and one or more leaves emerge
5	Embryo with evident root and two or more leaves (=seedling)

**Table 2:** Effect of different strengths of MS and VW media on seed germination and protocorm development of *P. amboinensis* J.J.Sm. within 10 weeks of culture.

Medium	Stage 0	Stage 1 (germination)	Stage 2 (protocorm)	Stage 3	Stage 4	Stage 5 (seedling)	Germination (stages 1–5)
MS	14.7 ± 2.3^d^	10.2 ± 1.2b^c^	21.4 ± 2.6^c^	21.1 ± 2.5^c^	19.7 ± 2.1^c^	12.9 ± 1.8^a^	85.3 ± 2.1^a^
1/2 MS	10.7 ± 2.3^c^	9.7 ± 1.4^b^	19.9 ± 2.1^b^	15.4 ± 2.1^b^	20.4 ± 2.3^c^	23.9 ± 1.6^b^	89.3 ± 2.1^b^
VW	2.7 ± 1.5^a^	5.1 ± 1.6^a^	12.9 ± 1.4^a^	13.5 ± 1.2^a^	14.4 ± 1.8^a^	51.4 ± 1.5^d^	97.3 ± 1.5^c^
1/2 VW	9.2 ± 1.5^b^	10.5 ± 2.6^c^	14.1 ± 2.3^a^	13.7 ± 2.1^a^	17.1 ± 1.5^b^	35.3 ± 2.1^c^	90.7 ± 2.8^b^

*Note*. Means ± standard errors followed by the same letter within a column are not significantly different based on Duncan's multiple range test at *P*=0.05.

**Table 3:** Effect of 15% (v/v) CW supplementation with various concentrations of banana homogenate (BH) on plantlet development of *P. amboinensis* J.J.Sm. within 10 weeks of culture.

Treatments	Root		Leaf			Plantlet	
	No.	Length (mm)	No.	Length (mm)	Max width (mm)	Length (mm)	Dry weight (g)
Control	6.2 ± 1.4^a^	24.1 ± 1.2^a^	3.8 ± 0.7^a^	24.3 ± 1.7^a^	10.1 ± 1.9^a^	41.6 ± 1.4^a^	11.8 ± 2.2^a^
CW 15% v/v	7.4 ± 1.2^ab^	31.2 ± 2.2^b^	6.5 ± 0.7^b^	36.3 ± 1.2^b^	12.2 ± 1.6^ab^	43.7 ± 1.2^a^	12.2 ± 1.1^a^
CW 15% v/v + BH 5 g·L^−1^	8.5 ± 1.1^b^	32.1 ± 2.1^b^	6.7 ± 0.8^b^	38.2 ± 1.3^b^	14.9 ± 1.2^b^	61.8 ± 0.9^b^	14.8 ± 0.9^b^
CW 15% v/v + BH 10 g·L^−1^	8.7 ± 1.4^b^	32.4 ± 2.4^b^	7.1 ± 1.3^b^	36.7 ± 0.9^b^	15.1 ± 1.1^b^	62.1 ± 1.3^b^	15.5 ± 1.4^b^
CW 15% v/v + BH 15 g·L^−1^	8.5 ± 1.1^b^	32.3 ± 2.1^b^	6.9 ± 1.4^b^	36.5 ± 0.7^b^	14.8 ± 0.8^b^	61.9 ± 1.2^b^	15.2 ± 1.3^b^

*Note*: Means ± standard errors followed by the same letter within a column are not significantly different based on Duncan's multiple range test at *P*=0.05.

## Data Availability

The data related of this article are available from the correponding author upon request.
